# Interruptions to Enteral Nutrition in Critically Ill Patients in the Intensive Care Unit

**DOI:** 10.7759/cureus.22821

**Published:** 2022-03-03

**Authors:** Maria Habib, Hafiz Ghulam Murtaza, Nusrat Kharadi, Tooba Mehreen, Anam Ilyas, Aimen H Khan, Moiz Ahmed

**Affiliations:** 1 Department of Critical Care Medicine, Shifa International Hospital Islamabad, Islamabad, PAK; 2 Department of Pulmonology, PAF Hospital Islamabad, Islamabad, PAK; 3 Department of Cardiology, National Institute of Cardiovascular Diseases, Karachi, PAK

**Keywords:** total parenteral nutrition, interruption of enteral nutrition, intensive care unit, enteral and parenteral nutrition, caloric deficit

## Abstract

Background

Malnourishment has been linked with increased morbidity and mortality among critically ill patients. The current study aimed to assess the factors contributing to the interruption of enteral nutrition so that preventive measures can be formulated to avoid the malnourishment of critically ill patients.

Methodology

A prospective, observational study was conducted at the Department of Intensive Care Unit, Shifa International Hospital, Islamabad, between November 2020 and May 2021. All patients admitted in the intensive care unit (ICU) during the study period aged between 18 and 80 years, who remained admitted in the medical ICU for at least 72 hours were included in the study. Those who had ileostomy or colostomy were excluded from the study. Diagnostic categories were defined as surgical and medical. Data on clinical parameters including admitting diagnosis and airway-related issues were recorded in a predefined proforma.

Results

The mean duration of enteral nutrition interruption in males was 13.96 ± 13.12 days while that of females, 12.48 ± 12.43 days. Non-invasive ventilation dependency was significantly associated with an interruption in enteral nutrition (p=0.002). The mean duration of interruption of enteral nutrition was not correlated with airway issues (p=0.569). However, the mean duration of interruption of enteral nutrition was significantly lower in patients who underwent spontaneous breathing trials with T-piece (p = 0.032). Those who were advised nil per oral before surgery had a significantly longer duration of enteral nutrition interruption (p < 0.0001) with a mean length of 30.18 ± 5.83 days. The duration of enteral nutrition interruption was significantly longer in patients who had tracheostomy than those who did not have a tracheostomy (26.3 ± 6.34 vs. 9.54 ± 11.61 days) (p < 0.0001).

Conclusion

The present study revealed that at least three-forth of the patients admitted in ICUs had documented orders to the interruption of enteral nutrition. The most significant causes that correlated with interruptions to enteral nutrition were non-invasive ventilation dependency, tracheostomy, spontaneous breathing trials with T-piece, and orders of nil per oral before surgery.

## Introduction

In critically ill patients, admitted to intensive care units (ICUs), interruptions to enteral nutrition are a common occurrence. Enteral feed is preferred over parenteral nutrition due to the obvious reason of total parenteral nutrition (TPN) therapy being complex; moreover, the high rate of associated complications and higher management cost [1,2]. In the past, it was considered that enteral nutrition can only be started once there is evidence of peristalsis, bowel movement, or flatus. Current evidence shows that enteral nutrition can be started within 48 hours of ICU admission and that too does not require the presence of bowel sounds or other signs previously mentioned [3-7]. The current guidelines recommend that the decision to interrupt enteral nutrition should only be done in order to carry out the diagnostic and therapeutic procedures [8]. Nevertheless, there are currently no protocols provided to prevent enteral nutrition interruptions.

There has been an established association between malnutrition and morbidity/mortality among critically ill patients [9,10]. A study by Tala, evaluated the significance of enteral nutrition, stating that a positive correlation between meeting protein needs and survival existed [11]. The authors suggested that the implication of a nutrition-focused model of care within the ICU will have a significant impact on patient outcomes. A significant proportion of patients admitted to medical ICU are at risk of malnutrition due to several reasons including poor nutritional status at baseline, catabolic state, and critical illness [7]. 

Enteral mode of feeding is the preferred mode of nutrition for critically ill patients; however, interruption of enteral nutrition in the ICU is not uncommon due to a number of reasons. Worth mentioning reasons for enteral feeding interruptions are feed intolerance and conducting diagnostic or therapeutic procedures. Uozumi et al. conducted research to know the causes for interruption of enteral feeding in ICU in their center. The authors revealed that 567 episodes of enteral nutrition interruption occurred over a period of 17.1 days while at least three interruptions were observed per patient. The main cause for enteral nutrition interruption was undetermined reasons in 29%, airway manipulation in 18%, gastrointestinal incidents in 14%, and dialysis in 13%. Surprisingly, only 12% of these episodes had clear written orders for interruption [12].

Keeping in mind the importance of enteral nutrition in critically ill patients and its relation to morbidity and mortality and owing to the scanty local literature we decided to study the reasons for interruption of enteral nutrition in our ICU. The objective of the study was to determine the most common reasons for interruption of enteral nutrition in ICU in order to highlight the emphasis on curtailing these interruptions to mitigate the risk of malnutrition in critically ill patients.

## Materials and methods

A prospective observational study was conducted at the Department of Intensive Care Unit, Shifa International Hospital, Islamabad, between November 2020 and May 2021. The ethical approval was procured from the institutional review board of Shifa International Hospital with reference # IRB/SIH/CCM/876. Informed consent was obtained from the patients or their attendants after narrating the clinical significance of the study in layman's terms.

A non-probability convenience sampling technique was used to perform participant recruitment. The sample size was determined using a reference study authored by Uozumi et al., who reported the highest rate of enteral feed interruption for undetermined reasons in 29% of patients [12]. Therefore, by keeping the sample proportion of 29%, a margin of error to be 6.5%, and a confidence level of 85%, a sample size of 101 was calculated.

All patients admitted in the ICU during the study period aged between 18 and 80 years, who remained admitted in the medical ICU for at least 72 hours were included in the study. Those who had ileostomy or colostomy were excluded from the study. Diagnostic categories were defined as surgical, medical, and other admissions.

Data were collected from the patient's medical files and included the notes for interruptions of enteral feeding. Data on socio-demographics including the age of the patient, gender, and clinical parameters including admitting diagnosis, use of prokinetic agents, procedures during the hospital stay, and airway-related issues were recorded in a predefined proforma.

All data were analyzed using Statistical Package for the Social Sciences (SPSS, IBM, Chicago, IL) version 26. Association between duration of enteral feeding interruption and sociodemographic and clinical parameters were assessed using chi-square test and t-tests where appropriate. A p-value of < 0.05 was set as the cutoff for statistical significance.

## Results

A total of 106 patients were included in the study with males constituting about 47.2% of the patients. Diagnosis at presentation was surgical in 30 (28.3%) and medical in the majority of the patients. Enteral nutrition interruption was documented in 78 (73.6%) patients. The remaining patient characteristics are illustrated in Table 1.

**Table 1:** Sociodemographic and clinical parameters of study participants (categorical variables)

Characteristics	N (%)
Gender	
Male	50 (47.2%)
Female	56 (52.8%)
Intermittent mandatory ventilation	
Yes	38 (35.8%)
No	67 (64.2%)
Admission diagnosis	
Surgical	30 (28.3%)
Medical	76 (71.7%)
Enteral nutrition interruption documented	
Yes	78 (73.6%)
No	28 (26.4%)
Prokinetic agents	
Yes	64 (60.4%)
No	42 (39.6%)
Bilevel positive airway pressure (BIPAP) dependent	
Yes	26 (24.5%)
No	80 (75.5%)
Airway-related issues	
Yes	39 (36.8%)
No	67 (63.2%)
Spontaneous breathing trial with T-piece	
Performed	9 (8.5%)
Not performed	97 (91.5%)
Nil per oral surgery	
Yes	17 (16%)
No	89 (84%)
Endoscopy	
Yes	24 (22.6%)
No	82 (77.4%)
Nil per oral tracheostomy	
Tracheostomy done	23 (21.7%)
Tracheostomy not done	83 (78.3%)
Nil per oral dialysis	
Dialysis done	19 (17.9%)
Dialysis not done	87 (82.1%)
Nil per oral intensive care units (ICU) procedure	
Yes	6 (5.7%)
No	100 (94.3%)

The mean age of patients was 61.83 ± 11.21 years with a mean duration for enteral nutrition interruption of 13.18 ± 12.72 days. The mean interval between a procedure and restarting the enteral feed for different causes is demonstrated in Table 2.

**Table 2:** Patient characteristics and clinical parameters (continuous variables)

Characteristics	Mean ± SD
Age (years)	61.83 ± 11.21
Enteral nutrition interruption episodes (in days)	13.18 ± 12.72
Caloric requirement (kcal/day)	1932.55 ± 180.74
Calories administered (kcal/day)	1560.66 ± 1903.58
Caloric deficit (kcal)	622.55 ± 250.5
Restart of diet after esophagogastroduodenoscopy (in days)	2.75 ± 0.52
Restart of diet after surgical procedure (in days)	1.29 ± 0.2
Restart of diet after airway (in days)	3.61 ± 0.17
Restart of diet after tracheostomy (in days)	5.85 ± 0.26
Restart of diet after surgical (in days)	6.31 ± 0.36
Restart of diet after dialysis (in days)	0.39 ± 0.1
Restart of diet after T-piece (in days)	2.48 ± 0.09

The mean duration of enteral nutrition interruption in males was 13.96 ± 13.12 days while that of females, 12.48 ± 12.43 days. Albeit, the difference was statistically insignificant. Bilevel positive airway pressure (BiPAP) dependency was significantly associated with an interruption in enteral nutrition. Those who were on BiPAP had a mean length of enteral nutrition interruption of 19.83 ± 14.16 (p=0.002). The mean duration of interruption of enteral nutrition was not correlated with airway issues (p=0.569). However, the mean duration of interruption of enteral nutrition was significantly lower in patients who underwent spontaneous breathing trials with T-piece (p=0.032). Those who were advised nil per oral before surgery had a significantly longer duration of enteral nutrition interruption (p < 0.0001) with a mean length of 30.18 ± 5.83 days. The duration of enteral nutrition interruption was significantly longer in patients who had tracheostomy than those who did not have a tracheostomy (26.3 ± 6.34 vs. 9.54 ± 11.61 days) (p < 0.0001) (Table 3). 

**Table 3:** Association of interruption of enteral nutrition with patient characteristics

Variables	Mean ± SD Enteral Nutrition Interruption Episodes (in days)	P-value
Gender		0.554
Male	13.96 ± 13.12	
Female	12.48 ± 12.43	
Intermittent mandatory ventilation		0.185
Yes	10.98 ± 11.03	
No	14.41 ± 13.5	
Admission diagnosis		0.21
Surgical	10.7 ± 11.69	
Medical	14.15 ± 13.05	
Enteral nutrition interruption documented		0.694
Yes	13.47 ± 12.78	
No	12.36 ± 12.75	
Prokinetic agents		0.895
Yes	13.04 ± 12.52	
No	13.38 ± 13.17	
Bilevel positive airway pressure (BIPAP) dependent		0.002
Yes	19.83 ± 14.16	
No	11.02 ± 11.5	
Airway-related issues		0.569
Yes	12.25 ± 12.44	
No	13.72 ± 12.94	
Spontaneous breathing trials with T-piece		0.032
Trial administered	4.49 ± 4.69	
No trial administered	13.98 ± 12.94	
Nil per oral before surgery		< 0.0001
Yes	30.18 ± 5.83	
No	9.93 ± 10.97	
Endoscopy		0.166
Yes	10 ± 6.93	
No	14.11 ± 13.87	
Nil per oral due to tracheostomy		< 0.0001
Tracheostomy performed	26.3 ± 6.34	
Tracheostomy not performed	9.54 ± 11.61	
Nil per oral due to dialysis		< 0.0001
Yes	3.54 ± 2.38	
No	15.28 ± 13.09	

## Discussion

The purpose of this study was to determine the reasons for interrupting enteral nutrition in ICU patients. We found several factors including dependency on non-mechanical ventilation, airway-related issues, and tracheostomy to be significantly correlated with the need to interrupt the enteral feed. The current study also indicated that the patients who underwent dialysis were negatively associated with interruptions to enteral feed. The previous study by Uozumi et al. revealed similar findings revealing that the mean duration of interruption to enteral nutrition in patients undergoing dialysis was six hours [12], while, in our study, it was almost four hours. The difference could be explained by the larger number of patients with intermittent dialysis in the former study.

Chittawatanarat et al. in their study found artichoke powder to lead to an increase in viscosity of enteral feeding due to its high fiber content [13]. No significant complications were noted in the study apart from three patients who had a high gastric volume. Salciute-Simene et al. discussed enteral nutrition to be interrupted during 35% of days of trial the main reasons being tracheostomy (16%), high gastric volume (17%), surgical interventions (16%), and hemodynamic instability (20%) [14]. The average duration of ENI was going to be 12 (6-24) hours, the longest ENI being due to factors related to patients often leading to underfeeding. Similarly, Lee et al. found 72% of enteral feeding interruptions to be due to related to procedures and avoidable human factors whereas only 20% were because of feeding intolerances [15]. The average duration of enteral feeding interruptions was three days and the total duration of stay in ICU was 24.5 hours.

The authors also concluded that a shorter duration was required to reach a minimum goal of 30 mL/kg/day after the early initiation of enteral feeding. Similar results were seen in studies conducted by Carvalhal et al. and White et al. [16,17]. White et al. studied the human enteral feeding transitions programs which were established to select patient groups which could be discharged with home enteral nutrition [17]. Home enteral nutrition after discharge from neonatal ICU was effective and safe along with 98% parent satisfaction and 10% increase in home enteral nutrition utilization (p=0.003) and a lower duration of stay (31.5 days). Orinovsky et al. in their study implemented early enteral feeding in the intervention group due to which intolerance was less in the intervention group as compared to the control group (p=0.03) after cessation of feeding [18]. Similarly, Savio et al. studied patients who were on mechanical ventilation in a prone position and received enteral nutrition with orogastric or nasogastric feeding which was well tolerated [19]. Furthermore, calories and protein given in the prone position were received effectively as compared to the supine position.

Like any other study, our study was also laden with a few limitations. The sample size was not large, which limited the generalizability of the study findings to a larger population. Further studies are required to assess the relationship between interruptions to enteral nutrition and its outcome in critically ill patients.

## Conclusions

The present study revealed that at least three-fourths of the patients admitted in ICUs had documented orders to the interruption of enteral nutrition. The most significant causes that correlated with interruptions to enteral nutrition were BiPAP dependency, tracheostomy, spontaneous breathing trials with T-piece, and orders of nil per oral before surgery. Further, large-scale and multi-center studies are required in order to ascertain the factors that can aid in reducing the duration of interruption of enteral feed in critically ill patients.
